# *In vitro* and *in vivo* evaluation of macromolecular prodrug GC-FUA based nanoparticle for hepatocellular carcinoma chemotherapy

**DOI:** 10.1080/10717544.2016.1264499

**Published:** 2017-02-21

**Authors:** Can Huang, Na-Mei Li, Pei Gao, Sa Yang, Qian Ning, Wen Huang, Zhi-Ping Li, Peng-Ju Ye, Li Xiang, Dong-Xiu He, Xiang-Wen Tan, Cui-Yun Yu

**Affiliations:** 1 Hunan Province Cooperative Innovation Center for Molecular Target New Drug Study, University of South China, Hengyang, China,; 2 Learning Key Laboratory for Pharmacoproteomics of Hunan Province, Institute of Pharmacy & Pharmacology University of South China, Hengyang, China, and; 3 Chemistry Department, Eastern Kentucky University, Richmond, KY, USA

**Keywords:** GC-FUA nanoparticles, 5-Fu, asialoglycoprotein receptor, drug delivery system, chitosan

## Abstract

A novel type of macromolecular prodrug delivery system is reported in this research. The N-galactosylated-chitosan-5-fluorouracil acetic acid conjugate (GC-FUA) based nanoparticle delivery system was evaluated *in vitro* and *in vivo*. Biocompatibility of GC-FUA-NPs was screened by BSA adsorption test and hemolysis activity examination *in vitro*. Cytotoxicity and cellular uptake study in HepG2 and A549 cells demonstrated that compared to free 5-Fu, the GC-FUA-NPs play great function in killing cancer cells for the cell endocytosis mediated by asialoglycoprotein receptor (ASGPR), which overexpresses on the cell surface. Pharmacokinetics study further illustrated that the drug-loaded nanoparticles has a much longer half-time than free 5-Fu in blood circulation in Sprague–Dawley (SD) rats. Tissue distribution was investigated in Kunming mice, and the result showed that the GC-FUA-NPs have a long circulation effect. The obtained data suggested that GC-FUA-NP is a very promising drug delivery system for efficient treatment of hepatocellular carcinoma.

## Introduction

Hepatocellular carcinoma (HCC) is the second most common cause of cancer-related deaths in the world, and it is one of the few cancers whose incidence has continually increased over the past decade (Zhan et al., 2016).

5-Fluorouracil (5-Fu), a chemotherapeutic drug and radiosensitizer (Kalantarian et al., 2010), is an effective chemotherapeutic agent which has been widely used in treating several malignant cancers, including liver, colon, breast, pancreas, ovarian and skin cancers, etc. (Tseng et al., 2015). 5-Fu is a cytotoxic drug, which has been used to treat cancers (Fadaeian et al., 2015) via inhibiting the synthesis of nucleic acids (Burns & Beland, 1983). However, several main side effects of chemotherapy have been recognized such as gastrointestinal reaction, cerebellar ataxia, low blood counts and myelosuppression (Alter et al., 2006; Wigmore et al., 2010). Another drawback of 5-Fu is the short plasma half-life of 10–20 min (Cheng et al., 2012). For overcoming these shortcoming, many researchers have been doing many try, with the development of nanotechnology in cancer therapy, the nanosystems of nanoparticles, liposomes, micelles are commonly applied in this field. In recent years, a promising approach to increase the aqueous solubility and enhance bioavailability of antitumor drugs is to form polymer-based prodrugs through the conjugation with polymeric carriers (Dragojevic et al., 2015; Magaña et al., 2016; Sun et al., 2016). The macromolecular prodrugs also possess other preponderance such as sustained drug release and reduced toxicity before the metabolization occurs (Yu et al., 2016). In this study, a novel strategy was utilized for one step *in situ* synthesis of a macromolecular pro-drug and the fabrication of an amphiphilic core–shell micelle (PDC-M). PDC-M shows a slow drug release and has a relatively high stability. In the basis of previous studies, 5-Fu, which has been prepared from macromolecule prodrug, was combined with chitosan (CS) by amide condensation reaction.

Chitosan is one of naturally occurring alkaline polysaccharides. CS nanoparticles offer many advantages because of their low toxicity, biodegradability and biocompatibility characteristics (Nagpal et al., 2010). Many studies have shown that asialoglycoprotein receptor (ASGPR), which highly expresses in the surface of hepatocytes, improved the targeting effect by endowing NPs with the active-targeting capacity (Kokudo et al., 2003; Tseng et al., 2014). Galactosylated chitosan (GC) is a galactose ligand. It has been recognized that it has a higher cytotoxicity towards cancer cells (HepG2) and a longer half-life in circulation system (Lou et al., 2016).

Furthermore, according to the literatures, the favorable permeability and good retention (EPR) effect are paramount for nanocarriers (including polymeric nanoparticles (Kawaguchi, 2000), liposomes (Zong et al., 2016), micelles (Sutton et al., 2007) and nanogels (Jung et al., 2008)) to accumulate in tumor tissues (Anitha et al., 2014; Bolkestein et al., 2016). Alternative formulations of free 5-Fu (such as microspheres, NPs, micelles and liposomes) (Udofot et al., 2015) could prolong retention time (Onishi & Machida, 2008; Huang et al., 2010; Li et al., 2014), reduce side effects (Wigmore et al., 2010; Noori et al., 2014) and improve bioavailability.

Furthermore, the *in vitro* cytotoxicity and cellular uptake, *in vivo* pharmacokinetic (PK) and tissue distribution of GC-FUA nanoparticles also were investigated in this research (our laboratory has been preparing a novel macromolecular prodrug N-galactosylated-chitosan-5-fluorouracil acetic acid (GC-FUA) conjugate based nanoparticle; Yu et al., 2015).

## Materials and methods

### Materials

Dulbecco’s modified Eagle’s medium (DMEM) and fetal bovine serum (FBS) were purchased from Gibco, Carlsbad, CA. MTT ([3-4,5-dimethylthiazol-2-yl]-2, 5-diphenylte-trazolium bromide) was purchased from Amresco (Solon, OH). HepG2 and A549 cells were purchased from Shanghai Life Sciences Academy, Shanghai, China. Kunming mice, Sprague–Dawley (SD) rats and New Zealand White rabbits were supplied by Laboratory Animal Center, University of South China, China. All other chemical reagents were of analytical grade and used without further purification.

### Preparation of GC-FUA nanoparticles

The preparation of the GC-FUA-NPs followed the established procedure, which has been described in detail elsewhere (Yu et al., 2015). The compound (GC-FUA) was synthesized by amide condensation reaction of GC and FUA. GC-FUA nanoparticle drug delivery systems (Yu et al., 2015) were fabricated in aqueous media (containing pH 4.5 and tripolyphosphate).

### Characterizations of GC-FUA nanoparticles

To characterize the prepared GC-FUA nanoparticles, the nanoparticle dispersion was firstly centrifuged at 25 °C for 10 min at 4000 rpm. Afterwards, the drug content in the supernatant was determined and considered as the drug loss in the preparation. The morphology of the GC-FUA nanoparticles was observed by transmission electron microscope (TEM, JEM-2100, Tokyo, Japan). Before observation, the sample was sputter coated with gold. The drug concentration of the GC-FUA nanoparticles was determined in triplicate by a Shimadzu UV-1750 UV-Vis spectrophotometer at 273 nm (Kyoto, Japan). Drug loading content is calculated through the following formula:

$$\mathrm{Drug}\;\mathrm{loading}\;\mathrm{content}\%\;=\frac{\mathrm{weight}\;\mathrm{of}\;\mathrm{the}\;\mathrm{drug}\;\mathrm{in}\;\mathrm{nanoparticles}}{\mathrm{weight}\;\mathrm{of}\;\mathrm{the}\;\mathrm{nanoparticles}}\times 100\%$$

The sizes and size distributions of GC-FUA nanoparticles were measured by a Zetasizer (Nano ZS, Malvern Instruments, Malvern, UK) before drug release.

### *In vitro* drug release study

GC-FUA and 5-Fu loaded GC (the control group) nanoparticles were sealed in a dialysis bag, and then incubated in 37 °C PBS (phosphate-buffered saline) pH 7.4 buffer with shaking at 100 rpm for 168 h. At predetermined time intervals (0.5, 1, 2, 4, 8, 12, 24, 48, 72, 120, 168 h), 3 ml sample was collected from the release medium and then the same amount of fresh PBS solution was added to the release medium. The drug concentration was then determined by measuring the absorbance at 273 nm in a Shimadzu UV-1750 UV-vis spectrophotometer (Kyoto, Japan). Test data were used to calculate the accumulated drug release.

### BSA adsorption

BSA (0.5 g) was dissolved in 1000 ml pH 7.4 PBS buffer. Five milliliters of the prepared BSA solution was added to 5-Fu and GC-FUA-NPs concentrated solutions (Log *C*= −1.39, −1.09, −0.79, −0.49, −0.19 mmol/l). The mixtures were vortexed for 5 min and then incubated at 37 °C in a water bath for different time periods of 1 h, 2 h, 4 h and 6 h. After the incubation, the samples were centrifuged at 15 000 rpm for 15 min, and each supernatant was stored separately. The BSA properties of 5-Fu and GC-FUA-NPs were investigated by spectrophotometry at 273 nm.

### Hemolysis activity examinations

To investigate the biocompatibility of GC-FUA-NPs, Six New Zealand White rabbits (half male and half female) were randomly selected. Five milliliter blood was drawn from their auricular vein and 0.2 ml anticoagulant was then added. The mixture was washed in PBS followed by centrifuge (800 rpm) to concentrate the red blood cells. This process was repeated several times until the supernatant was no longer red. A 2% red blood cell suspension was prepared by PBS dilution. All samples were stored at −4 °C until analysis. 0.5 ml GC-FUA-NPs and free 5-Fu at different concentrations were mixed separately with 0.5 ml of the red blood cell suspension. The mixtures were incubated at 37 °C in a water bath for three hours and centrifuged at 3000 rpm for 10 min. Afterwards, 100 μl of the supernatant of each sample was loaded into a 96-well plate. Optical density was measured by the absorbance at 540 nm on a microplate reader (Labsystems Multiskan, Bio-chromatic Labsystem, Osaka, Japan). PBS and distilled water were used as negative and positive controls, respectively. The hemolysis ratio of red blood cells was calculated with the following equation.

$$\mathrm{Hemolysis}\;\mathrm{ratio}\;\left(\%\right)=\frac{\mathrm{Sample}-\mathrm{negative}\;\mathrm{control}}{\mathrm{Positive}\;\mathrm{control}-\mathrm{negative}\;\mathrm{control}}\;\times \mathrm{optical}\;\mathrm{density}\times 100\%$$

### Cytotoxicity and cellular uptake of nanoparticles *in vitro*

#### Cytotoxic viability assay

As reported in the literature, HepG2 cells were found with overexpressed ASGPR on the cell surfaces and A549 cells were not found to express ASGPR (Yang et al., 2010). The cells were incubated in DMEM medium supplemented with 10% FBS and 1% ciprofloxacin solution at 37 °C, 5% CO_2_. The mixture was digested with 0.25% trypsin and washed three times with PBS buffer. HepG2 and A549 cells were sealed into 96-well plates, 5 × 10^3^ cells cellular density per well with three replicate wells for each concentration. The cells were incubated for more than 24 h and then treated with 5-Fu and GC-FUA-NPs concentration of 5, 1.7, 0.56, 0.19, 0.63 and 0.021 mmol/l, respectively. Twenty-four and 48 h later, the drug-treated cells were washed twice with PBS and incubated for four hours in 20 μl 5 mg/ml MTT in the medium. After the culture medium was removed, cells were then lysed in 150 μl of DMSO. Optical density was measured by the absorbance at 570 nm on a microplate reader (Labsystems Multiskan, Bio-chromatic Labsystem, Osaka, Japan). Inhibition rate was calculated using the following equation.

$$\mathrm{Inhibition}\;\mathrm{rate}\;\left(\%\right)=\frac{\mathrm{Control}\;\mathrm{group}-\mathrm{drug}\;\mathrm{group}}{\mathrm{Control}\;\mathrm{group}}\;\;\;\;\;\times \mathrm{optical}\;\mathrm{density}\times 100\%$$

The IC_50_ (50% inhibitory concentration) value was determined by running the statistical software SPSS 18.0 (Chicago, IL).

#### Cellular uptake assay

HepG2 (A549) cells were plated at a density of 1 × 10^6^ cells per well into six-well plates, with three replicate wells for each amount to be used. The medium was changed every two days. When the cells in a six-well plate increased to 80–90%, Hanks' balanced salt solution (HBSS; pH 7.4) was used to replace the before medium. Cell balancing for two hours was then mixed with fresh HBSS to 1 ml (0.5 mmol/l) FUA and GC-FUA-NPs were incubated. Two hours later, the cells were washed three times with pre-cooled HBSS, followed by addition of 200 μl of distilled water. All samples were stored at −80 °C until analysis. The sample was applied to freeze–thaw cycles three times. Subsequently, they were centrifuged at 12 000 rpm for 15 min and dispensed with the supernatant. The 200 μl supernatant was transferred to a new tube, and acceded to internal standard (5-bromouracil at 50 μg/ml). The mixture was vortexed for 5 min and then centrifuged at 4 °C for 10 min at 12 000 rpm. FUA content was analyzed on an Agilent 1260 HPLC system as plasma sample (Santa Clara, CA).

#### Targeting effect assay

Galactose can combine with ASGPR, thereby competitively inhibit the binding of GC-FUA-NPs with ASGPR under the same conditions. HepG2 and A549 cells were plated at a density of 1 × 10^6^ cells per well into six-well plates, with three replicate wells for each dosage form. Once cells reached 80–90% confluence in six-well plates, the medium was replaced with HBSS containing 1 mg/ml of galactose. The rest of the operation has been described in Cellular uptake assay section.

### Pharmacokinetics (PK) and tissue distribution of nanoparticles *in vivo*

#### Pharmacokinetic analysis

Six rats were randomly divided into two treated groups. Various formulations of 5-Fu (group 1: free 5-Fu; group 2: GC-FUA-NPs) were injected into the tail veins of SD rats at a dosage of 35 mg/kg (Yu et al., 2014), and the blood was collected from rats by the fossa orbitalis vein into heparinized tubes at selected time periods of 0.25, 0.5, 1, 2, 4, 8, 12, 24 and 48 h, and centrifuged for 10 min at 4000 rpm to separate plasma. Ethyl acetate was used to isolate 5-Fu from the plasma. Afterwards, 200 μl of plasma and 1 ml ethyl acetate were acceded into a 2 ml Eppendorf (EP) tube, in which 50 μl of the 50 μg/ml 5-bromouracil internal standard was added subsequently. The mixture was vortexed for 5 min and then centrifuged for 15 min at 4000 rpm. The supernatant was transferred to a new clean EP tube, and residues were nitrogen dried at room temperature. The dry residues were later dissolved in 100 μl of mobile phase (the mixture of 5% acetonitrile and 95% water) followed by vortex-mixing for 1 min. The mixture was centrifuged for 10 min at 12 000 rpm and the supernatant was analyzed by Agilent 1260 HPLC system (Santa Clara, CA). All samples were stored at −20 °C until analysis. Pharmacokinetic parameters were obtained by running the software PK Solver 2.0.

#### *In vivo* biodistribution analysis in Kunming mice

Fifty four mice were divided into two groups with each group 27. Various formulations of 5-Fu (group 1: free 5-Fu; group 2: GC-FUA-NPs) were injected into the tail veins of mice at a dosage of 35 mg/kg. Mice were sacrificed by cervical dislocation after 0.5, 1, 2, 4, 8, 12, 24, 48 and 72 h injection, and then dissected. Tissues including heart, liver, spleen, lung and kidney were suctioned with a filter paper, accurately weighed, and homogenized in 2.0 ml of saline (0.9% NaCl). Tissues homogenates were repacked (1 ml) and internal standard mix (50 μl) was taken in a 5 ml tube. Ethyl acetate extraction was used to separate 5-Fu from the tissues homogenate. The mixture was vortexed for 1 min and then pretreated with 2 ml of ethyl acetate. After another 5 min vortex-mixing and centrifugation for 10 min at 4000 rpm, the organic layer was transferred to 2 ml EP tube and evaporated with a gentle stream of nitrogen gas at 37 °C. The dry residues were dissolved in 100 μl of the mobile phase followed by two-minute vortex-mixing, and a 20 μl aliquot of the sample was injected into the HPLC system for analysis. All samples were stored at −80 °C until analysis.

### Chromatographic conditions

The chromatographic system (an Agilent 1260 system, Santa Clara, CA) consists of a quaternary pump, a manual injector, and UV detector using Agilent Chemstation as acquisition and data analysis software (Agilent Technologies, Santa Clara, CA). Analyses for samples were carried out on a 250 mm × 4.6 mm (5 μm) Hypersil C18 column; the column temperature was set at 30 °C. The mobile phase consists of acetonitrile and distilled water with a volumetric ratio of 5/95 (v/v). The flow rate was 0.6 ml/min and detection was set at 265 nm.

## Result and discussion

### Preparation and characterization of GC-FUA nanoparticles

Chitosan is the only natural positively charged polymer that can bind anions, such as CO_3_^2−^. GC-FUA nanoparticles were prepared by ionic crosslinking method (Carrillo et al., 2014; Yu et al., 2015). With the addition of pH = 4.5 TPP solution, the interaction was mediated by the electrostatic forces between the protonated NH_3_^+^ groups and the negative charged residues in TPP. One mg/ml of TPP was used to avoid the formation of precipitate in the preparation of nanoparticles. Drug-loading content was determined to be 21.25 ± 2.3% (*n* = 3).

**Figure 1.**
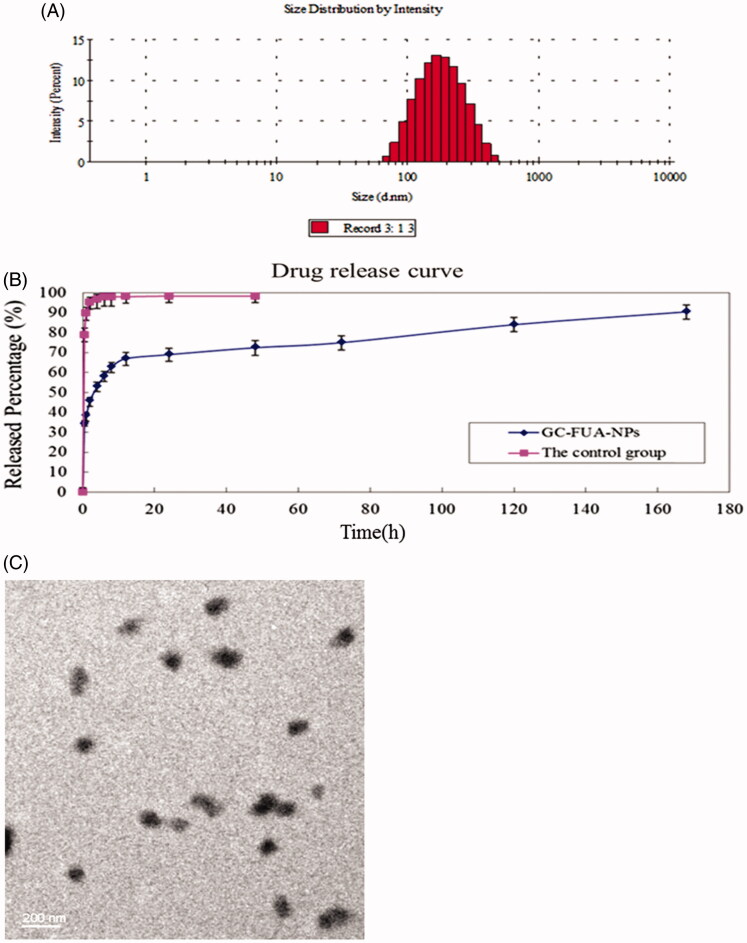
Size distribution of GC-FUA-NPs (A), and release profile of the GC-FUA-NPs and 5-Fu loaded GC nanoparticles in PBS (*n* = 3, B), and transmission electron microscope (TEM) image of GC-FUA nanoparticles (C).

As depicted in Figure 1(A), the size of GC-FUA nanoparticles ranged from 100 to 300 nm in a normal distribution and the mean value was about 163.2 nm. TEM images can be seen in Figure 1(C) and show that the GC-FUA nanoparticles were particulate matter of nanoscale size. The result indicated the above preparation method could offer good size control of the nanoparticles (Yu et al., 2009).

UV analysis showed the drug release profile of FUA of GC-FUA NPs in PBS buffer (pH = 7.4) at 37 °C. As shown in Figure 1(B), the control group, 5-Fu loaded GC nanoparticles achieved approximately 100% drug release within four hours. In contrast, the GC-FUA-NPs were released slowly, with a cumulative release percentage of 95.4% after seven days. The results indicated GC-FUA NPs could elongate the treatment time as a carrier of 5-Fu.

### BSA adsorption

**Figure 2.**
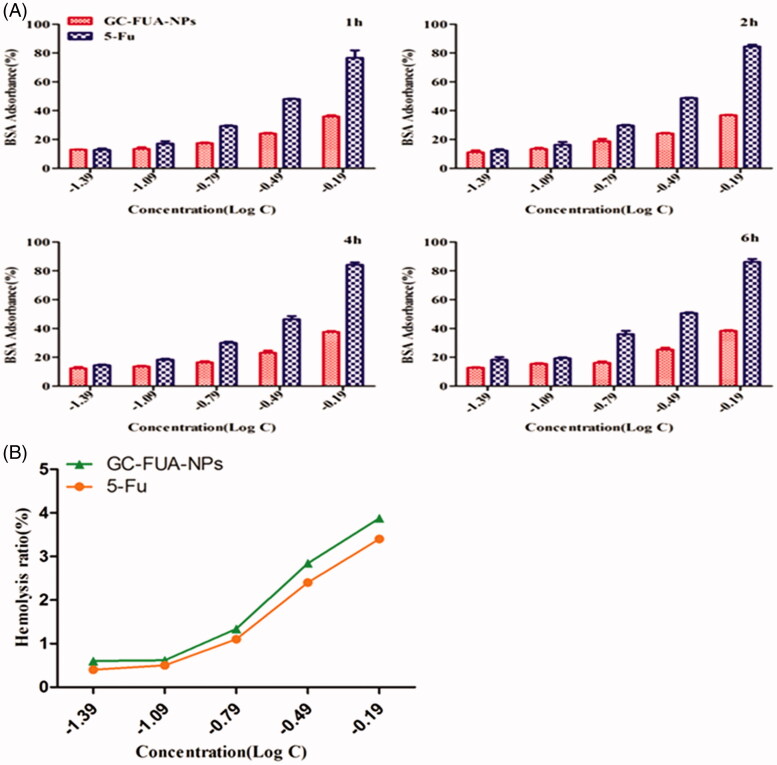
BSA adsorbance incubated with GC-FUA-NPs and free 5-Fu at different times (A), and percentage of red blood cell hemolysis incubated with GC-FUA-NPs (B).

Figure 2(A) shows the BSA adsorption of GC-FUA-NPs at different concentrations for 1, 2, 4 and 6 h. Compared with free 5-Fu, the GC-FUA-NPs showed much lower BSA adsorption. In addition, with the increase in concentration, the difference between the two groups became larger. More than 80% of BSA adsorbance to 5-Fu remains at a log concentration of −0.19 g/l, whereas only 34% of adsorption was observed after treatment with free 5-Fu at the same concentration. The present rate of GC-FUA-NPs did not increase at a certain time. GC-FUA-NPs exhibited low adsorbance and could be used as a biocompatible delivery carrier, and give a good biocompatibility.

### Hemolysis activity examination

The nano-sized GC-FUA-NPs, designed to be administered via intravenous injection for most drug delivery applications, need to be biocompatible and safe. The percentage of hemolysis caused by the GC-FUA-NPs at different concentrations is depicted in Figure 2(B). It is observed that the percent hemolysis ratio increased with the increase of the concentration of GC-FUA-NPs. All the GC-FUA-NPs with a concentration above 650 μg/ml (Log *C*= −0.19 μg/ml) exhibit hemolysis below 5% (Li et al., 2012), which show their permissibility.

### Cytotoxic activity assay (MTT)

**Figure 3.**
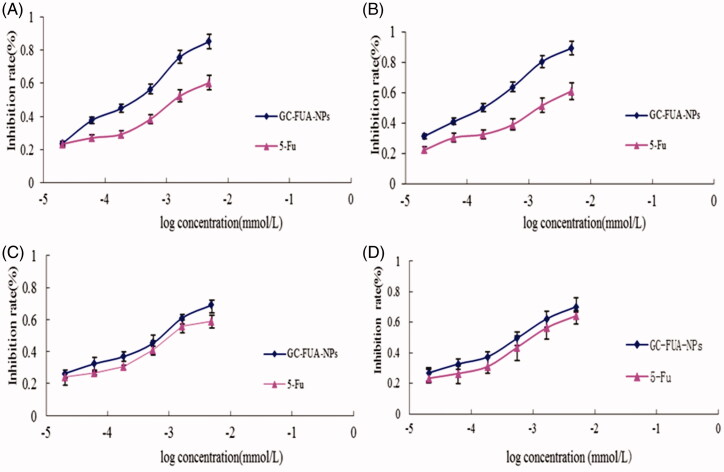
*In vitro* cell inhibition of 5-Fu and GC-FUA-NPs in HepG2 at different times (A) 24 h and (B) 48 h, and in A549 at different times (C) 24 h and (D) 48 h (*n* = 3, *p* > 0.05 versus 5-Fu).

In MTT assay, the HepG2 and A549 were treated by GC-FUA nanoparticles. In order to investigate whether drug-loaded nanoparticles can be used as a potent anticancer agent, the cytotoxic activity of 5-Fu and GC-FUA-NPs was further investigated in the same cell line *in vitro*. From Figure 3, it could be seen that the GC-FUA-NPs could significantly inhibit the proliferation of HepG2 in a dose and time-dependent manner, and are more efficient than free 5-Fu to kill HepG2 (*p* < 0.05). Although the GC-FUA-NPs showed obvious inhabitation of the proliferation of A549 cells as well, its effect is insignificant compared to the free 5-Fu (*p* > 0.05). 5-Fu, Table 1(A) summarizes the IC50 values of 5-Fu and GC-FUA-NPs for HepG2 and A549 cells. Table 1 shows that the IC50 value of GC-FUA-NPs (0.238 mmol/l) is decreased by 0.463 mmol/l in HepG2 cells at 24 h compared to free 5-Fu (0.701 mmol/l); in A549 cells, the IC50 value of GC-FUA-NPs (0.498 mmol/l) is 0.174 mmol/l less than free 5-Fu (0.672 mmol/l); After 48 h, the IC50 values of GC-FUA-NPs (0.147 mmol/l) is reduced by 0.515 mmol/l in HepG2 cells compared to free 5-Fu (0.662 mmol/l); the IC50 value of GC-FUA-NPs (0.433 mmol/l) was shown to be 0.168 mmol/l lower than free 5-Fu (0.601 mmol/l) in A549 cells. The difference could stem from the fact that ASGPR could be mediated through cell endocytosis and this could make it easier for GC-FUA-NPs to enter into the tumor cells compared to free 5-Fu group. Therefore, the GC-FUA-NPs could be more efficient to kill tumor cells with similar mechanisms of 5-Fu and improve the targeted anticancer effect.

**Table 1.** IC_50_ of 5-Fu and GC-FUA-NPs in HepG2 and A549 cells (A), and pharmacokinetic parameters of 5-Fu and GC-FUA-NPs administration to SD rats (*n* = 3, B).

A			
		IC_50_ (mmol/l)	
Drug	Time(h)	HepG2	A549
5-Fu	24	0.701 ± 0.023	0.672 ± 0.042
	48	0.662 ± 0.022	0.601 ± 0.037
GC-FUA nanoparticles	24	0.238 ± 0.021^a^	0.498 ± 0.035
	48	0.147 ± 0.017^a^	0.433 ± 0.031
B			
Parameter	5-Fu	GC-FUA-NPs	
*T*_1/2_ (h)	0.55	40.55	
AUC_0–_*_*t*_* (μg/ml·h)	153.99	326.91	
AUC_0–∞_ (μg/ml·h)	155.31	651.00	
CL (μg/(μg/ml)/h)	44.00	11.46	
*V*_SS_ (μg/(μg/ml))	41.62	528.43	

^a^*p* < 0.05 versus 5-Fu.

### Cellular uptake assay

**Figure 4.**
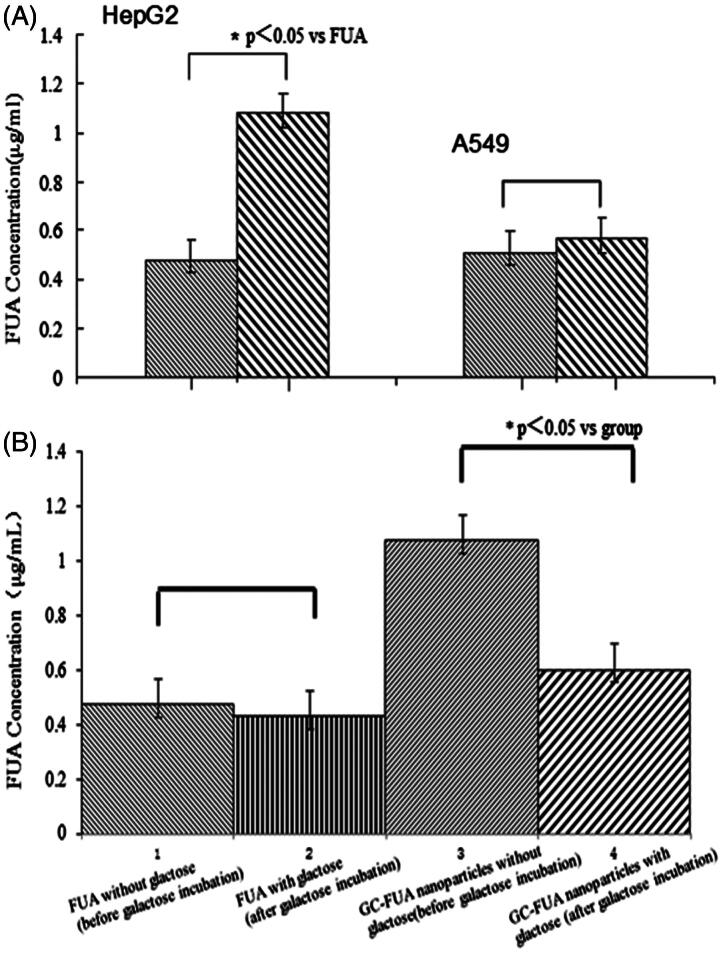
*In vitro* cell uptake of the FUA and GC-FUA-NPs against (*n* = 3, A), and galactose incubation cells uptake of the FUA and GC-FUA-NPs by HepG2 and A549 cells (*n* = 3, B).

The cellular uptake of the samples is shown in Figure 4(A) on the basis of HPLC measurement. Under the chromatographic conditions mentioned above, two kinds of cells (HepG2 and A549 cells) were investigated for their uptake of FUA and GC-FUA-NPs. Overall, HepG2 cells uptake of GC-FUA-NPs system is 1.26 times higher than that of free FUA (*p* < 0.05). However, there is no significant difference between GC-FUA-NPs and free FUA taken up by A549 cells. The results suggest that GC-FUA-NPs system could function as a grafted galactosyl which could specifically recognize ASGPR on HepG2 cell surface, and accelerate its uptake by the inner-mediated endocytosis.

### Targeting effect

In order to further illustrate targeting effect, exogenous galactose effect to HepG2 cell uptake was investigated. We first added the galactose solution (65 μg/ml) in HepG2 cells with ASPGR receptor binding site pre-saturated for the competitive inhibition. As shown in Figure 4(B), there was no significant difference (*p* > 0.05) in the cellular uptake of FUA compared to galactose saturated FUA under the same conditions. Nevertheless, notable difference was observed between GC-FUA-NPs system and free FUA about 0.56-folds (*p* < 0.05). The overall results confirmed the targeting efficiency of galactosylated-chitosan-based nanoparticles towards HepG2 cells mediated by ASGPR.

### *In vivo* pharmacokinetics analysis

**Figure 5.**
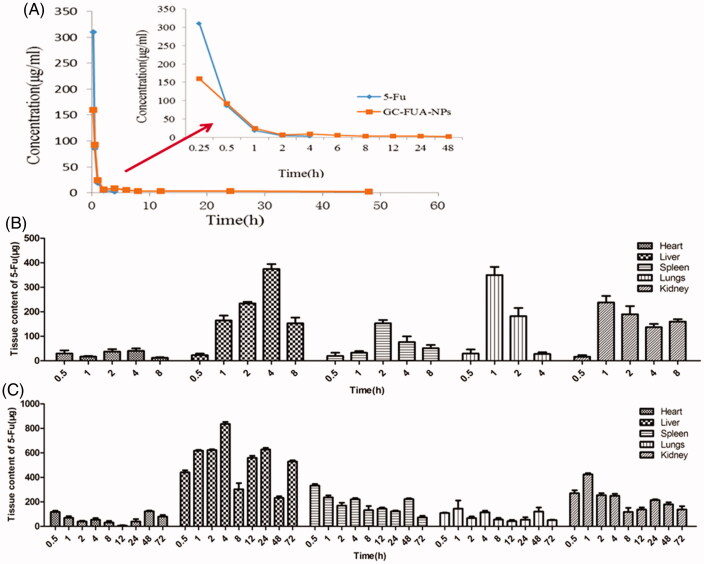
Mean 5-Fu concentration–time curves in plasma after tail vein i.v. injection of free 5-Fu and GC-FUA-NPs to SD rats (*n* = 3, A), and biodistribution of free 5-Fu (B) and GC-FUA-NPs (C) in tissue (*n* = 3).

The concentration of 5-Fu in plasma was determined by HPLC. The 5-Fu concentration–time curves in plasma after intravenous administration of free 5-Fu and GC-FUA-NPs to rats are shown in Figure 5(A). It showed that the GC-FUA-NPs exhibited significant PK advantages compared to free 5-Fu. The GC-FUA-NPs evidently display prolonged PK character over free 5-Fu. Free 5-Fu was not able to be detected in the plasma four hours after tail vein injection. However, the concentration of 5-Fu released by GC-FUA-NPs was still detectable (2.26 μg/ml) in the plasma after 48 h. This means that the N-galactosylated-chitosan-based nanoparticles could slowly release 5-Fu *in vivo*. The peak concentration of the free 5-Fu group was much higher than the GC-FUA-NPs group, suggesting that GC-FUA-NPs could extend drug release and reduce side effects compared to free 5-Fu. The PK parameters were acquired by running the PK Solver 2.0 software and are summarized in Table 1(B). Half-life (*T*_1/2_) generally refers to the time required for a quantity of drug to reduce to half of its initial value. The half-life of the GC-FUA-NPs was found to be 40.55 h and is 72.73 times longer than free 5-Fu (0.55 h). The total area under the curve (AUC_0–∞_) values of the formulation was 4.19-folds higher than that of free 5-Fu. Moreover, AUC_0–_*_t_* values of free 5-Fu are 153.99 μg/ml·h lower than that of GC-FUA-NPs. This result demonstrated that the bioavailability of GC-FUA-NPs was apparently improved compared to that of free 5-Fu.

The steady-state volume of distribution (*V*_ss_) is an important PK parameter to evaluate drug disposition in body blood. Calculation by the software (PK Solver 2.0) showed the GC-FU-NPs volume of distribution was 528.43 μg/(μg/ml), which is 11.7 times higher than that of free 5-Fu (*V*_ss_=41.62 μg/(μg/ml)). All data indicate the GC-FUA-NPs as a promising drug delivery system for targeted and sustained drug release mediated by ASGPR.

### *In vivo* biodistribution analysis in normal mice

Herein, the content of 5-Fu in various tissues after tail vein intravenous injection with free 5-Fu or GC-FUA-NPs at a dose of 35 mg/kg is presented in Figure 5(B). Apparently, the highest content of 5-Fu was found in liver. GC-FUA-NPs were specifically recognized by ASGPR, a part of which can be rapidly metabolized by the liver’s dihydropyrimidine dehydrogenase (DPD enzyme). The kidneys are the main organs of 5-Fu excretion. In four hours, the content of 5-Fu in liver reached the maximum (818.97 μg/ml), which is much higher than that of other tissues. With increased time, the content of 5-Fu in various organs decreased dramatically in both 5-Fu and GC-FUA-NPs. After eight hours, no 5-Fu could be detected in the free 5-Fu group while it was still detectable in the GC-FUA-NPs group at organs except liver and kidney after 72 h. This demonstrated that the GC-FUA-NPs have prolonged cycling effect compared to free 5-Fu.

## Conclusions

In summary, the results of the present study indicated that a novel strategy could be used to fabricate GC-FUA nanoparticles, which function as drug delivery system. GC-FUA-NPs showed excellent biocompatibility in the BSA adsorption and hemolysis activity examinations. *In vitro* cytotoxicity and cellular uptake studies showed that the GC-FUA-NPs with galactose residues could specifically recognize ASGPR receptors on HepG2 cell surface, and generated lower cytotoxicity than free 5-Fu. Pharmacokinetics evaluation in SD rats and tissue bio-distribution in Kunming mice confirmed that GC-FUA-NPs have much longer half-life than free 5-Fu in the circulation system. Although future research would be needed in order to improve the effect of GC-FUA-NPs in BALB/c nude mice bearing HCC mass and other mouse model, GC-FUA-NPs have shown great potential as a treatment for liver tumor. Future study would focus on the synergistic antitumor effect of multifunctional nanoparticles, which could offer better drug-loading content and greater antitumor effect *in vitro* and *in vivo*.
